# Prevalence and pattern of rheumatic heart disease in the Nigerian savannah: an echocardiographic study

**Published:** 2007-07

**Authors:** Mahmoud U Sani, Kamilu M. Karaye, Musa M. Borodo

**Affiliations:** Department of Medicine, Aminu Kano Teaching Hospital and Department of Medicine, Bayero University, Kano, Nigeria; Department of Medicine, Aminu Kano Teaching Hospital and Department of Medicine, Bayero University, Kano, Nigeria; Department of Medicine, Aminu Kano Teaching Hospital and Department of Medicine, Bayero University, Kano, Nigeria

## Abstract

**Background:**

Rheumatic heart disease (RHD) remains a major public health problem in developing countries. Whereas Africa has 10% of the world’s population, as many as half of the 2.4 million children affected by RHD globally live on the continent. RHD accounts for a major proportion of all cardiovascular disease in children and young adults in African countries. While acute rheumatic fever is on the decline even in the developing world, there are still a large number of chronic rheumatic heart disease cases, often complicated by chronic congestive heart failure and recurrent thrombo-embolic phenomena, both posing greater challenges for management. We report on the prevalence and pattern of valve involvement in RHD using echocardiography from our centre.

**Methods:**

In this retrospective study, transthoracic echocardiography (TTE) data collected from two echocardiography laboratories in Kano over a period of 48 months (June 2002 to May 2006) were reviewed. Patients with a diagnosis of rheumatic heart disease were selected. Information obtained from the records included the age, gender, clinical diagnosis and echocardiographic diagnoses.

**Results:**

A total of 1 499 echocardiographic examinations were done in the two centres over the four-year study period. One hundred and twenty-nine of the 1 312 patients (9.8%) with abnormal results had an echocardiographic diagnosis of RHD. There were 47 males and 82 females (ratio 1:1.7) and their ages ranged from five to 60 (mean 24.02 ± 12.75) years. Mitral regurgitation was the commonest echocardiographic diagnosis present in 49 patients (38.0%). Thirty-six (27.9%) patients had mixed mitral valve disease, 25 (19.5%) had mixed aortic and mitral valve disease, 10 (7.8%) had pure mitral stenosis and four (3.1) had pure aortic regurgitation. Complications of RHD observed included secondary pulmonary hypertension in 103 patients (72.1%), valvular cardiomyopathy in 41 (31.8%), and functional tricuspid regurgitation was seen in 39 (30.2%).

**Conclusion:**

Our data show that RHD is still an important cause of cardiac morbidity and a large proportion of the patients already had complications at diagnosis. There is an urgent need to implement the ASAP programme of the Drakensberg declaration to avert this scourge.

## Summary

Rheumatic heart disease (RHD) remains a major public health problem in developing countries. Whereas Africa has 10% of the world population, as many as half of the 2.4 million children affected by RHD globally live on the continent.^1^ RHD accounts for a major proportion of all cardiovascular disease in children and young adults in African countries and, by extension, in the world, because 80% of the world’s population live in developing countries where the disease is still rampant. The disease has the potential to undermine national productivity since it affects the most productive part of the population.^1^

The major determinants of acute rheumatic fever (ARF) and RHD are poverty, malnutrition, overcrowding, poor housing and a shortage of healthcare resources. Although cost-effective strategies for the prevention and control of these diseases are available, they remain under-utilised in most developing countries.

According to the World Health Organisation (WHO), RF/RHD affects about 15.6 million people worldwide, with 282 000 new cases and 233 000 deaths each year.^2^ There are 2.4 million affected children between five and 14 years of age in developing countries, one million of whom live in sub-Saharan Africa, making the continent the major RF/RHD hotspot.^3^ Up to 1% of schoolchildren in Africa, Asia, the eastern Mediterranean region and Latin America show signs of RHD.^4^ There are about two million people with RHD requiring repeated hospitalisation and one million likely to require surgery globally.^4^ However, these estimates are based on conservative assumptions, so the true disease burden is likely to be substantially higher. Furthermore, the overall quality of epidemiological data from developing countries is poor, particularly with respect to research documenting the incidence of ARF.^2^

Over the past century, as living conditions have become more hygienic and less crowded, and nutrition and access to medical care have improved, ARF and RHD have become rare in developed countries. The introduction of antibiotics has also helped to reduce the burden of disease, although to a lesser extent than these other factors.^5^,^6^ ARF and RHD are now largely restricted to developing countries and some poor, mainly indigenous populations of wealthy countries.

While acute rheumatic fever is said to be on the decline even in the developing world, we are burdened with a large number of chronic rheumatic heart disease cases. These patients exhibit chronic congestive heart failure and recurrent thrombo-embolic phenomena, both posing greater challenges for management. We set out to describe the prevalence and echocardiographic pattern of RHD in Kano, the most populous city in the Nigerian savannah region.

## Methods

In this retrospective study, transthoracic echocardiographic (TTE) data collected over a 48-month (June 2002 to May 2006) period was reviewed. Patients with an echocardiographic diagnosis of rheumatic valvular heart disease were selected. The study was carried out at the echocardiography laboratory of Aminu Kano Teaching Hospital and at a private echocardiography laboratory, both located in Kano, northern Nigeria. The two laboratories are the only places were the test was done during the study period and they serve all the echocardiography requests in the State in addition to receiving referrals from three neighboring states. Kano, the study site, is the most populous state in the Nigerian savannah region with a population of 9.38 million people (2006 National Census).

Information obtained from the records included age, gender, names of referring hospital/physician, clinical diagnosis and echocardiographic findings. Data were analysed using SPSS version 10.0 software.

## Technical information

The echocardiography machine used at Aminu Kano Teaching Hospital, Kano, Nigeria was ATL HDI 1500, bought by the Federal Government of Nigeria from ATL Ultrasound PO Box 3003 Bothell, WA 98041-3003 USA. The echocardiography machine used at the private laboratory was ATL Ultramark 9, also bought from the USA (refurbished). The two machines are maintained by Philips Project Company (PPC) limited, Abuja, Nigeria.

Echocardiographic modalities applied included M-mode, two-dimensional (2D) and Doppler studies. Echocardiography was done with 3–5 MHz sector transducer. Complete 2D echocardiographic examination was performed according to the recommendations of the American Society of Echocardiography (ASE).^8^ M-mode echocardiograms were derived from 2D images. The M-mode cursor on the 2D scan was moved to specific areas of the heart to obtain measurements, according to the recommendation of the committee on M-mode standardisation of the ASE.^9^ Doppler indices of left ventricular (LV) diastolic filling were obtained. Complete Doppler study was done according to the recommendations of the ASE.^10^

From the M-mode measurements, indices of LV function were derived. These included shortening fraction, ejection fraction, left ventricular mass, cardiac output and relative wall thickness. Echo examinations included valvular architecture; left atrial size; left ventricular size and function; a semi-quantitative estimate of the severity of valvular regurgitation; size and function of the right ventricle; and evidence of pulmonary arterial hypertension.

Mitral stenosis was diagnosed on the presence of thickening, diastolic doming, and restriction of leaflet motions. In severe cases, calcification, fibrosis, and limited leaflet excursion and fusion of commisures and chordae tendinae were also identified. Mitral regurgitation was diagnosed in the presence of thickened valves, dilated mitral valve annuli, and left atrial and left ventricular dilatation and lack of coaptation of the mitral valve leaflets in systole. Doppler echocardiographic analyses identified the presence and severity of regurgitation of the aortic, mitral and tricuspid valves.

Thickened and calcified aortic valve leaflets with reduced leaflet motion (aortic cusp separation less than 9 mm) suggested aortic stenosis. In addition, there could be concentric left ventricular hypertrophy (LVH) and decreased LV ejection performance. Aortic regurgitation was diagnosed when echocardiography with Doppler interrogation of the aortic valve showed the spatial extent of the colour Doppler aliasing in the outflow tract and was used as a rough guide of the severity of aortic insufficiency.^11^ A semi-quantitative scale of mild, moderate and severe insufficiency was predicated primarily on the area of the jet of disturbed flow in the outflow tract and, to a lesser extent, on the depth to which the jet penetrated toward the apex of the left ventricle.

The presence of vegetations was considered to suggest infective endocarditis. This was, however, limited by the fact that TTE could only detect vegetations ≥ 5 mm in diameter.^12^ Valvular cardiomyopathy was characterised by ventricular dilation, with normal or decreased wall thickness and diminution in systolic function (defined as ejection fraction less than 40%)^13^ in the presence of established valvular disease. Typically, defects that produce volume-overloaded states (regurgitation) are more likely to cause cardiomyopathy than lesions associated with pressure overload (valvular stenosis).

Echocardiographic findings associated with pulmonary hypertension included dilated pulmonary artery and dilation and hypertrophy of the right ventricle (RV), diastolic flattening of the interventricular septum and Doppler evidence of pulmonary hypertension.^14^

## Results

**Fig. 1.**
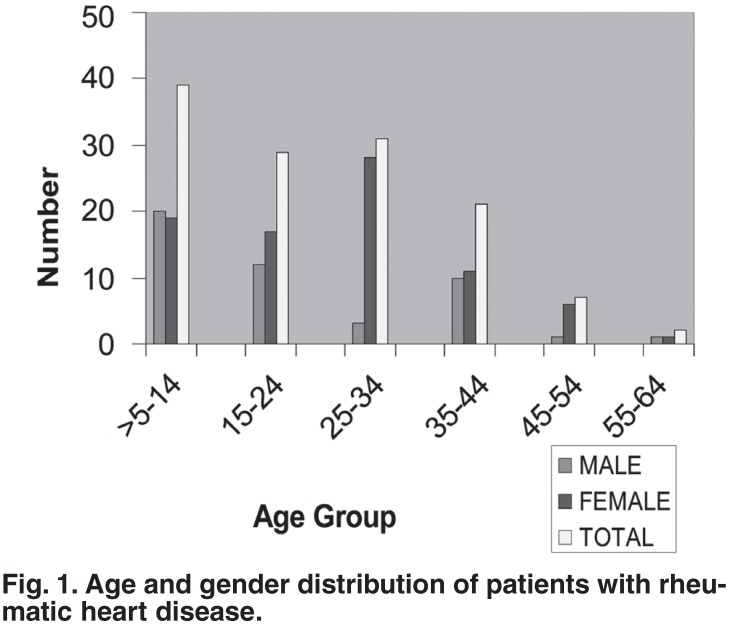
Age and gender distribution of patients with rheumatic heart disease.

A total of 1 499 echocardiographic examinations were done in the two centres over the four-year study period. Of these, 1 312 (87.5%) patients had an abnormal echocardiogram. One hundred and twenty-nine of the 1 312 (9.8%) patients had echo diagnosis of RHD. There were 47 males and 82 females (ratio 1:1.7) and their ages ranged from five to 60 (mean 24.02 ± 12.75) years. Fig. 1 shows the age and gender distribution of the study subjects. The highest prevalence was in the five to 14-year age group. Two patients were in their fifth decade.

The main clinical indications for echocardiography included RHD, 79 patients (61.6%); dilated cardiomyopathy, 10 (7.8%); hypertensive heart disease, 10 (7.8%); congestive cardiac failure of unknown cause, nine (7.0%); cerebrovascular accident, five (3.9%); congenital heart disease, five (3.9%); peripartum cardiac failure, four (3.1%); transient ischaemic attack, one (0.78%); and hypertrophic cardiomyopathy, one (0.78%). The test was requested by a wide range of medical practitioners, including general practitioners, physicians (non-cardiologists) and a cardiologist. The accuracy of a clinical diagnosis of RHD in this study was 61.6%.

**Fig. 2.**
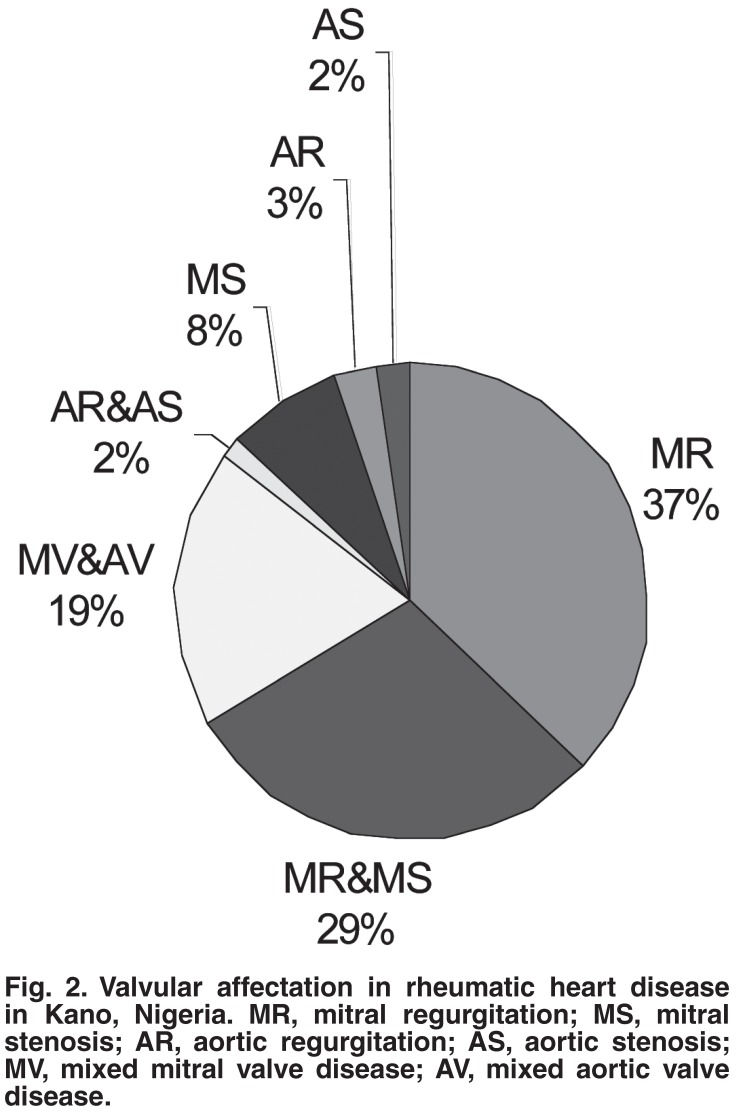
Valvular affectation in rheumatic heart disease in Kano, Nigeria. MR, mitral regurgitation; MS, mitral stenosis; AR, aortic regurgitation; AS, aortic stenosis; MV, mixed mitral valve disease; AV, mixed aortic valve disease.

Mitral regurgitation was the commonest echocardiographic diagnosis present in 49 (38%) of the patients, followed by mixed mitral valve disease in 36 (27.9%), and then combined mitral valve and aortic valve disease in 25 (19.5%) patients. The distribution of the various valvular diseases according to gender is shown in Table 1. Fig. 2 shows the valvular involvement among the patients studied.

**Table 1:** The Distribution Of The Various Valvular Diseases According To Gender In Kano, Nigeria

*Valve lesion*	*Male*	*Female*	*Total*	*Percentage*
Mitral regurgitation	17	32	49	38.0
MV disease	6	30	36	27.9
MV and AV disease	15	10	25	19.5
Mitral stenosis	4	6	10	7.8
Aortic regurgitation	2	2	4	3.1
Aortic stenosis	2	1	3	2.3
Mixed AV disease	1	1	2	1.6
Total	47	82	129	100

MV, mixed mitral valve; AV, mixed aortic valve.

Table 2 shows the complications of rheumatic heart disease seen at echocardiography. One hundred and three (72.1%) patients already had secondary pulmonary hypertension, a third of which was severe. The second and third commonest complications observed were valvular cardiomyopathy and functional tricuspid regurgitation, seen in 41 (31.8%) and 29 (22.5%) patients, respectively.

**Table 2:** Complication Of RHD Seen At Echocardiography In Kano, Nigeria

*Complication*	*Number*	*Percentage*
Pulmonary hypertension	103	72.1
Valvular cardiomyopathy	41	31.8
Functional TR	39	30.2
Infective endocarditis	11	8.5
Atrial fibrillation	13	10.1
Left ventricular thrombus	3	2.3

TR, tricuspid regurgitation.

## Discussion

Poverty, malnutrition, overcrowding, poor housing and a shortage of healthcare resources are still prevalent in Africa, including Nigeria. These are the major determinants of acute rheumatic fever and chronic rheumatic valvular heart disease. RHD was found to account for 9.8% of cardiac disease found at echocardiography in this study, similar to the 9.2% found in Port Harcourt, Nigeria.^15^ It was also close to the 11% found by Freers *et al.* among 500 patients referred for echocardiography in Uganda.^16^ The findings of this study, however, differed from those of Hakim *et al.* working in Zimbabwe, who found 25.1% of 1 153 patients referred for echocardiogram had RHD.^17^

All these findings are much higher than the WHO documented RHD prevalence of 5.7/1 000 patients in sub-Saharan Africa.^2^ This is probably because all four studies considered all age groups, whereas the WHO prevalence was among children five to 14 years old. In addition, these studies were echocardiography based; such referred patients have a high chance of having an abnormal heart, hence the high prevalence. This study was performed in the Nigerian savannah zone and ARF and RHD have been documented to be more common in the dry savannah grasslands than in the rainforest,^18^ probably because *Streptococcus pyogenes* is carried easily in the dry air and because of overcrowding among the poor during the colder seasons.

In many populations, ARF and RHD are more common in females than males.19 This has also been shown in this study. Whether this trend is a result of innate susceptibility, increased exposure to group A streptococcus because of greater involvement of women in child rearing, or reduced access to preventive medical care for girls and women is unclear.

In this study 61.6% of the patients were suspected to have rheumatic heart disease clinically. This shows the high index of suspicion for the disease at this stage. At the ARF stage when chronic valvular damage can be prevented by adequate antibiotic treatment, ARF is hardly considered by many doctors in developed countries.^20^ In the developing countries where it remains a daily challenge, there is still poor physician/patient awareness of ARF with low penicillin usage,^3^ militating against control of ARF and prevention of chronic RHD.

Mitral regurgitation was the commonest echocardiographic diagnosis, either alone in 49 (38%) of the patients, or in combination with mitral stenosis in 36 (27.9%) or aortic valve disease in 25 (19.5%). This is in agreement with the findings of local studies^21^-^23^ in Nigeria as well as those in other parts of the world.^17^,^24^ Combined mitral regurgitation and mitral stenosis was the second commonest abnormality in 27.9% of patients, similar to the findings of other workers.^22^,^24^ Pure mitral stenosis was seen in 7.8% of the patients, much lower than the 26% document by Jaiyesemi *et al.* among children.^21^ The reason for this could not be ascertained. Involvement of the aortic valve alone was less common. Most patients with aortic valve disease had it in combination with mitral valve disease. Organic tricuspid valve and pulmonary valve disease were rare. It is known that the tricuspid valve is rarely affected by rheumatic carditis,^25^ but functional tricuspid regurgitation may accompany mitral valve disease. This was seen in 39 (30.2%) of the patients in this study.

Pulmonary hypertension, possibly a result of walking and activity, which is inevitable for poor people, develops early and is usual in patients with mitral valve disease. One hundred and three (72%) of our patients had this complication. Secondary pulmonary hypertension in the early phase is due in large part to reactive changes in the pulmonary vascular resistance. In mitral and aortic valve disease, the left ventricle becomes hypertrophic and less distensible, leading to increased LV end-diastolic pressure. This causes increased work of the left atrium (LA), leading to hypertrophy and enlargement of the atrium. The output of the left atrium decreases and pulmonary hypertension results.

LA enlargement frequently results from mitral valve and to a lesser extent aortic valve disease. Atrial fibrillation (AF) may complicate LA enlargement and was seen in 13 (10%) of our patients in this study. AF leads to loss of organised mechanical activity of the LA and increases the tendency to develop spontaneous echo contrast and thrombus. None of our patients had left atrial thrombus. This might have been understated as TTE has limitations in detecting the presence of left atrial thrombi, and transoesophageal echocardiography (TEE) is more valuable, especially if thrombi occur in the left atrial appendage.^26^

Valvular cardiomyopathy is a specific dilated cardiomyopathy produced by valvular defects of numerous aetiologies. Typically, defects that produce volume-overloaded states (regurgitation) are more likely to cause cardiomyopathy than lesions associated with pressure overload (valvular stenosis). Forty-one (31.8%) of the patients in this study had this complication, most likely because more of the patients had valvular regurgitation than stenosis. It is associated with decreased LV systolic function with stasis and a predisposition to thrombus formation. LV thrombus was found in three (2.3%) of the patients, two of whom had an infarctive stroke.

The finding of vegetations in patients with established valvular heart disease suggested infective endocarditis. Eleven (8.5%) of the patients had vegetations, all of which were attached to the mitral valve. RHD is a known predisposing condition to the development of infective endocarditis. It has been shown to be the pre-existing condition for 66% of cases of infective endocarditis in two studies in different parts of Nigeria.^27^27,^28^

This study has shown that RHD was responsible for up to 10% of cardiac morbidity among patients referred for echocardiography in our environment. Most of the patients were children and young adults, many of whom already had severe disease at presentation, requiring surgical intervention. Balloon valvuloplasty, valve repair or replacement require expert teams in tertiary-care centres.^29^

There is a lack of primary, secondary and tertiary (medical and surgical treatment) programmes in Nigeria and many other countries in sub-Saharan Africa.^30^ While cost-effective strategies for the prevention (primary and secondary) and treatment (or tertiary prevention) of RF/RHD are available, the primary, secondary and tertiary prevention of RF and RHD are woefully inadequate in almost all African countries.^1^ Concerted efforts to control RF/RHD must be bolstered as soon as possible in the developing world so that progress can be made towards eradicating what is an entirely preventable disease.^31^

The Awareness, Surveillance, Advocacy, Prevention (ASAP) proposal is a comprehensive programme for the control of RF and RHD that was adopted at the first All-Africa Workshop on Rheumatic Fever and Rheumatic Heart Disease in the Drakensberg, South Africa, convened by the Pan-African Society of Cardiology (PASCAR).^32^ The objective for developing ASAP was to create a simple, modular but comprehensive model for RF/RHD control in Africa, based on interventions of proven efficacy, which could be adopted in part or in total by national departments of health or non-governmental organisations with a commitment to reducing the burden of disease attributable to RF/RHD in Africa.^31^

This programme entails raising the awareness of the public and healthcare workers with regard to RF and RHD; improving the quality of information available on the incidence, prevalence and burden of RF/RHD through epidemiological surveillance; working together as advocates to change public policy for the improvement of the healthcare facilities needed to treat and prevent the disease; and working towards the establishment of national primary and secondary prevention programmes for RF and RHD. This programme will be co-ordinated throughout Africa by the PASCAR in collaboration with the World Heart Federation and the World Health Organisation.

## Conclusion

Rheumatic heart disease is still an important cause of morbidity in our environment. There is an urgent need for every country in Africa to implement a national ASAP programme of the Drakensberg declaration to avert this scourge.
